# Surgical Management of Ascending Aortic Aneurysm and Its Complications

**DOI:** 10.1155/2014/102605

**Published:** 2014-06-26

**Authors:** Sisira Sran, Manpreet Sran, Nicole Ferguson, Amgad N. Makaryus

**Affiliations:** ^1^Department of Medicine, Nassau University Medical Center, 2201 Hempstead Turnpike, East Meadow, NY 11554, USA; ^2^NYIT-COM, Nassau University Medical Center, East Meadow, NY, USA; ^3^New York Institute of Technology, College of Osteopathic Medicine, Northern Boulevard, P.O. Box 8000, Old Westbury, NY 11568-8000, USA; ^4^Department of Cardiology, NuHealth, Nassau University Medical Center, 2201 Hempstead Turnpike, East Meadow, NY 11554, USA

## Abstract

Ascending aortic aneurysms involving the proximal aortic arch, arising anywhere from the aortic valve to the innominate artery, represent various problems in which open surgery is generally required. Surgical options include excision of the aortic pathology or wrapping the aneurysm shell with an aortic Dacron graft. Intervention using the latter method can lead to extravasation of blood along the suture lines resulting in continuous bleeding within the periprosthetic space. The Cabrol technique was developed as a method for decompression of postoperative leaks by the formation of a conduit system from the periprosthetic space to the right atrium. The coronary ostia are anastomosed to a second graft in an end-to-end fashion, which is then anastomosed to the ascending aortic conduit side to side. The native aorta is then sewn around the prosthesis, hereby creating a shunt to drain anastomotic leakage. This shunt reduces postsurgical risk of pseudoaneurysm formation and normally closes a few days following surgery. We discuss the case of a patient who underwent Cabrol's variation and six months later was demonstrated to have a patent shunt.

## 1. Introduction

The etiology of ascending aortic aneurysms is most likely multifactorial including increasing age, hypertension, tobacco use, genetics, atherosclerosis, and connective tissue disorders [1]. Risk factor reduction is an important goal of aortic aneurysm treatment along with medical therapy or various surgical options. The main treatment options for ascending aortic aneurysms involving the ascending aorta and aortic root associated with aortic valve disease include Classic Bentalls operation, the Cabrol variation, and Button Bentalls procedure.

## 2. Case Presentation

**Figure 1:**
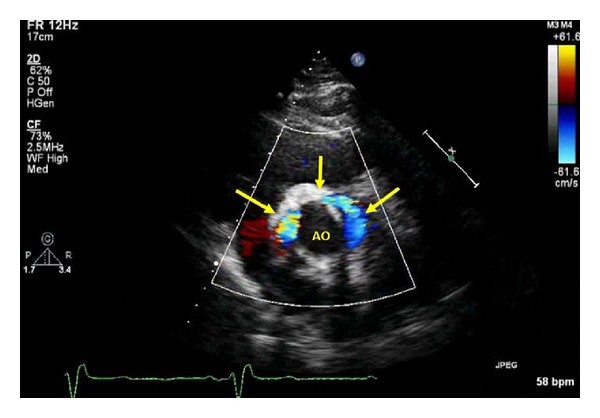
Transthoracic echocardiography revealing a shunt between the aortic root and the right heart involving the right atrium.

**Figure 2:**
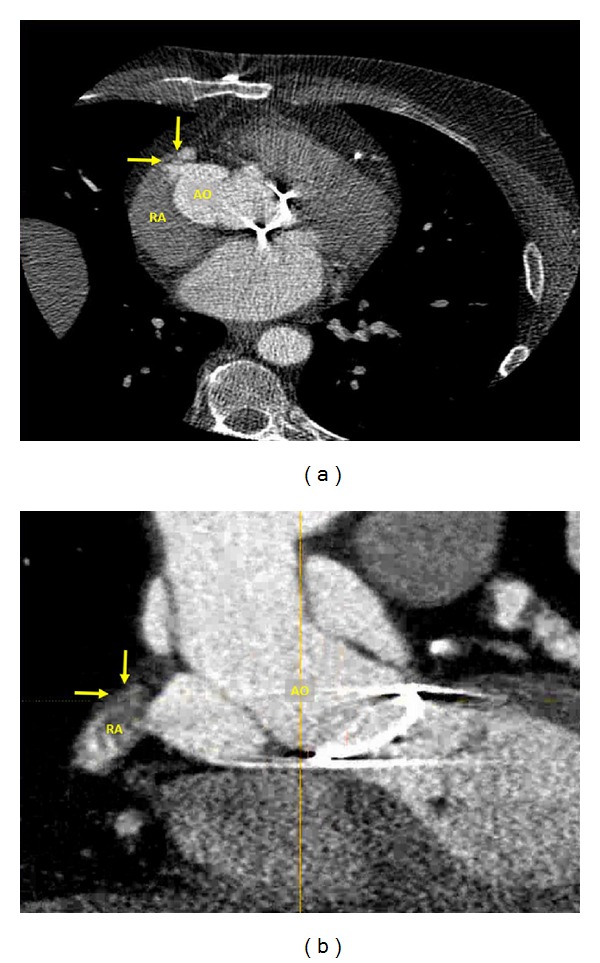
Cardiac CT scan demonstrates a contrast shunt within the wrapped portion of aorta with the right atrium during ventricular systole.

A 63-year-old man was referred for cardiothoracic surgery after the diagnosis of severe aortic regurgitation secondary to an ascending aortic aneurysm involving the aortic root. The patient was noted to have a dilated ventricle with global hypokinesis and a left ventricular ejection fraction estimated at 45%. The patient underwent surgery with placement of a St. Jude valve conduit (Bentall operation) and recovered well after surgery with no complaints. Approximately 6 months later, he presented complaining of progressive shortness of breath. A transthoracic echocardiography was obtained which revealed a shunt between the aortic root and the right heart involving the right atrium (Figure 1). Shunt fraction (Qp : Qs) was measured to be ~1.1. The decision was made to obtain a cardiac CT to assess the graft and thoracic aorta. In the CT scan (Figures 2(a) and 2(b)), a contrast shunt was demonstrated within the wrapped portion of aorta with the right atrium during ventricular systole. This represents a patent Cabrol shunt (placed at surgery), which should have spontaneously closed. Being that the shunt was small with Qp : Qs ~ 1.1 without right sided dilation, the patient was treated conservatively. Our conservative management included monitoring and close follow-up of the patient as he preferred not to have any further operative procedures.

## 3. Discussion

Ascending aortic aneurysms involving the proximal aortic arch, arising anywhere from the aortic valve to the innominate artery, represent various problems and open surgery is usually required. The Classic Bentall procedure involves replacement of the aortic valve and ascending aorta with reimplantation of the coronary arteries using a composite graft, association of a mechanical valve, and the use of a tube graft. The Cabrol technique involves anastomosing the coronary ostia to a second graft in an end-to-end fashion, which is then anastomosed to the ascending aortic conduit side to side. It includes oversewing the native aorta around the prosthesis and creating a shunt into the right atrium to drain anastomotic leakage from the periprosthetic space [2]. The shunt is expected to reduce the postsurgical risk of pseudoaneurysm formation and closes within a few days after surgery [3]. The button method requires mobilization of the coronaries and fashion of ostial “buttons” which are then attached to the graft. This method can be utilized when the disease process has severely impacted the root of the aorta.

Although aortic root and thoracic aortic surgical techniques have continued to improve, death from postoperative hemorrhage remains at 5 to 10% [4]. The Cabrol variation is preferred when dealing with catastrophic postoperative hemorrhage [3, 5]. Cabrol and colleagues commented that the periprosthetic-to-right atrial fistulas usually close soon after the operation [6]. The persistence of the fistula is most likely due to suture line dehiscence resulting in increased blood flow preventing the fistula from closing [7]. If the fistula becomes hemodynamically problematic, however, it can be surgically closed relatively easily [8]. With most shunts closing during the first postoperative week, late complications would be similar to those reported while using open techniques; however, in the small minority of patients with patent Cabrol shunts for longer periods, complications still need to be researched further. Few persistent patent Cabrol shunts have been reported; Tosoratti and colleagues discussed a patient with a valve conduit detachment and patent surgical fistula 16 years after the procedure presenting with heart failure and severe pulmonary hypertension [9]. These few cases demonstrate that a patent Cabrol shunt should be included on the differential diagnosis for any patient presenting with a past history of cardiac surgery, a left-to-right shunt, and a continuous murmur [7].

The Button Bentall method is the current recommended option with the classic Bentall and Cabrol variation used for special circumstances. Long-term mortality of the Button Bentall method was shown to be related to embolism and bleeding events similar to valve surgery. Contaminant CABG also increased bleeding risk for patients undergoing the Bentall procedure [10]. Midulla and colleagues comment that late mortality in patients is often due to a subsequent aneurysm or dissection along the aorta even in patients not presenting with a genetic defect affecting aortic structure. Frequent follow-up and control of risk factors such as hypertension are required postoperatively [11]. The button modification is preferred as it provides superior results with a hospital mortality of 4%, fewer reoperations, and excellent long-term survival [12].

Choice of method depends on various factors. The Cabrol method should be used when reduction and decompression of a false lumen is required. Most surgeons have now adopted aortic root replacement with direct coronary artery implantation to reduce pseudoaneurysm formation at the coronary and the aortic anastomoses [13]. Because the diseased aorta is completely removed, there is no option to wrap the graft with the aneurysmal shell or aortic wall, as is done in the Bentall operation. Surgical treatment of ascending aortic aneurysms depends on the extent of involvement of surrounding structures. If the width of the aortic valve leaflets, the annulus, and the sinuses of Valsalva are normal, then the aneurysm is replaced with a simple supracoronary Dacron tube graft. If the aortic valve is diseased but the aortic sinuses and annulus are normal then a Wheat procedure is performed. This involves replacing the aortic valve separately and replacing the aneurysm with a supracoronary synthetic graft.

In conclusion, our case shows a surgical complication of an ascending aortic aneurysm. Although most Cabrol shunts will close within the first postoperative week, symptomatic patients should be monitored for signs suggesting continued patency of the shunt, and all patients should have frequent follow-up. Our patient demonstrated a small shunt which was able to be conservatively treated, although some cases may require further surgery.
